# Advanced Neuropsychological Diagnostics Infrastructure (ANDI): A Normative Database Created from Control Datasets

**DOI:** 10.3389/fpsyg.2016.01601

**Published:** 2016-10-20

**Authors:** Nathalie R. de Vent, Joost A. Agelink van Rentergem, Ben A. Schmand, Jaap M. J. Murre, ANDI Consortium, Hilde M. Huizenga

**Affiliations:** ^1^Department of Psychology, University of AmsterdamAmsterdam, Netherlands; ^2^Department of Medical Psychology, Academic Medical CenterAmsterdam, Netherlands; ^3^Amsterdam Brain and Cognition Center, University of AmsterdamAmsterdam, Netherlands; ^4^See full list of consortium members on www.andi.nl; ^5^Research Priority Area Yield, University of AmsterdamAmsterdam, Netherlands

**Keywords:** normative comparisons, aggregate data, regression-based norms, Box-Cox transformation

## Abstract

In the Advanced Neuropsychological Diagnostics Infrastructure (ANDI), datasets of several research groups are combined into a single database, containing scores on neuropsychological tests from healthy participants. For most popular neuropsychological tests the quantity, and range of these data surpasses that of traditional normative data, thereby enabling more accurate neuropsychological assessment. Because of the unique structure of the database, it facilitates normative comparison methods that were not feasible before, in particular those in which entire profiles of scores are evaluated. In this article, we describe the steps that were necessary to combine the separate datasets into a single database. These steps involve matching variables from multiple datasets, removing outlying values, determining the influence of demographic variables, and finding appropriate transformations to normality. Also, a brief description of the current contents of the ANDI database is given.

## 1. Introduction

An important element of neuropsychological practice is to determine whether a patient who presents with cognitive complaints has abnormal scores on neuropsychological tests. In the diagnostic process, a number of neuropsychological tests are administered and the test results of the patient are compared to a normative sample, that is, a group of healthy individuals which resemble the patient in characteristics unrelated to the suspected disease or trauma. In this manner, a clinician can determine whether the patient's test scores should be interpreted as abnormal, and whether or not the patient may have a disorder.

Traditionally, scores are compared to normative data published in the manuals of the neuropsychological tests. However, there are a number of limitations associated with this approach. First, normative data of neuropsychological tests might have become outdated and no longer represent the patients we see today (Strauss et al., 2006). Second, many published tests lack norms for the very old population (80+) (Whittle et al., 2007). Third, some tests do not come with norms at all and clinicians have to make do with norms from other countries or with norms they themselves have gathered (Crawford and Garthwaite, 2002). Fourth, normative scores from test manuals are often only corrected for age but not for other demographic variables, such as level of education or sex (Lezak et al., 2012). Fifth, normative data are typically collected for one test at a time, as part of its construction, and standardization process. As a result, mostly univariate but not multivariate data are available. Recent studies have shown that multivariate normative comparison methods are more sensitive to deviating profiles of test scores than multiple univariate analyses (Crawford and Garthwaite, 2002; Huizenga et al., 2007; Castelli et al., 2010; Schmand et al., 2010; Smeding et al., 2011; Valdés-Sosa et al., 2011; González-Redondo et al., 2012; Broeders et al., 2013; Cohen et al., 2015; Su et al., 2015). Moreover, new univariate methods for normative comparisons, that use a resampling technique, require multivariate normative data as well (Huizenga et al., 2016).

Because of the limitations outlined above, we started the Advanced Neuropsychological Diagnostic Infrastructure project (www.andi.nl^1^). Our goal was to overcome these limitations by creating a large database from a demographically diverse group of healthy participants who completed several neuropsychological tests. This database will be accompanied by an interactive website where clinicians and researchers can upload their patients' scores. Interactive software on the website compares the patients' scores to demographically corrected norm scores from the database using advanced multivariate and univariate methods (Huizenga et al., 2007, 2016). The ANDI database and accompanying website will simplify normative comparisons and will provide more sensitive and specific normative comparisons.

In this article, we describe the step-by-step procedure of the ANDI normative database construction, so that the procedure can be replicated in other countries and in other fields of study that also rely on normative comparisons, such as clinical psychology or personnel psychology. We also describe current contents of the ANDI database. Finally, we address the advantages and potential limitations of the ANDI database in comparison to other normative data.

We illustrate these steps using Rey's Auditory Verbal Learning Test (AVLT) (Rey, 1958), an internationally well-known test. It is one of the tests that are also included in the ANDI database. The AVLT measures memory and learning (Strauss et al., 2006; Lezak et al., 2012). In its simplest form participants are presented with a list of 15 nouns, which they are asked to reproduce immediately (in any order). This is repeated five times. Twenty minutes after the five learning trials, there is a delayed recall condition in which participants are asked again which words they remember. Finally, there is a multiple choice recognition condition.

## 2. Construction of the ANDI database

**Figure 1:**
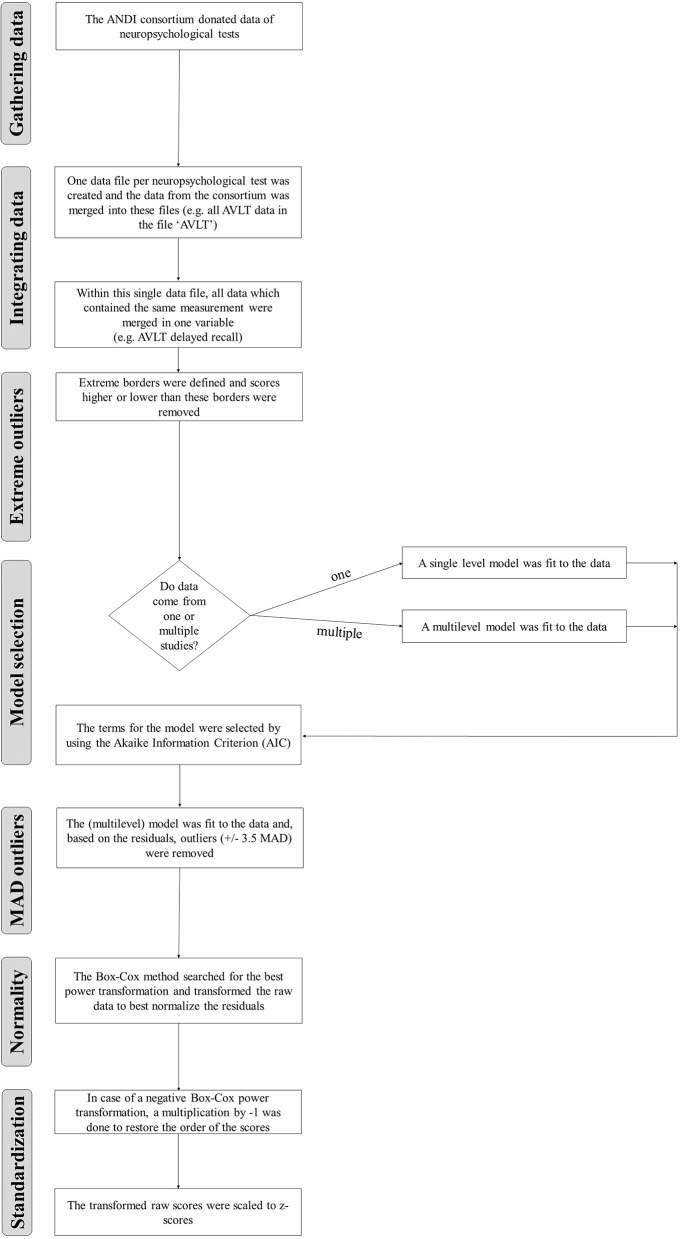
**Flow chart describing all steps of the database construction**.

For every neuropsychological test variable included in the ANDI database, a standardized automatized stepwise procedure was followed. A flow chart summarizing all steps can be found in Figure 1. In the following paragraphs, we explain the rationale for the steps and how they were applied.

### 2.1. Gathering data

The first step was to collect a large amount of normative data on neuropsychological tests. In cooperation with a group of researchers, the “ANDI consortium” (see www.andi.nl for a list of members) was created. The consortium members donated data of healthy control subjects, which they had collected in predominantly clinical research projects. All studies were approved by local ethics committees. All participants had sufficient knowledge of the Dutch language to complete the tests. All data were anonymized and could not be traced back to individual participants.

Example: Data on the (Rey) Auditory Verbal Learning Test (AVLT) from 32 research projects were donated, yielding data from a total of 5121 participants.

### 2.2. Integrating data

We created separate files for all neuropsychological tests. Each file contained multiple test variables. Also, the demographic variables age, sex, and level of education, were included for each participant. Only cases with scores on all three demographic variables were included. For each study a unique study identifier was added.

Example: One file for the AVLT was created. In this file data from all test variables were collected. Thus the variable AVLT-1 contained all data on the first trial of the AVLT, the variable AVLT-2 contained data on trial 2, and so on.

### 2.3. Removing impossible scores

After merging the data, we checked whether all scores were valid. Invalid scores might be coding errors, or deviant scores observed only in patients with severe pathology. If such invalid scores would not be removed from the database, the variance in scores would be overestimated, which would cause a diminished sensitivity to detect impairments. However, we also wanted the database to be an accurate representation of variability in the healthy population. This implied that the removal criteria should not be too strict. First, we removed the most extreme values. These were scores that were either due to an administrative error or could not come from a healthy participant. For every variable of each neuropsychological test, upper and lower “extreme borders” were defined. The upper border was set at the maximum possible score. This removed administrative errors. The lower border was set at the worst score a participant can obtain while still deemed cognitively healthy. To this end, we selected the raw score corresponding to the lowest published percentile of the worst performing normative sample. The exact percentile depended on the resolution of the published norm table, but generally a score corresponding to the first percentile was selected. Thus, for a test that has declining scores with increasing age, the raw score that was obtained from the lowest percentile of the oldest participants was defined as the lower extreme border. If no information from manuals was available, which fortunately was the case for a small number of tests, we asked members of the ANDI consortium to provide acceptable borders. On average 0.48% of scores were removed for the 183 variables. All extreme borders can be found in the ANDI background documentation (www.andi.nl).

Example: The upper border of the AVLT delayed recall is 15. Scores above 15 are impossible and thus were removed. The lower border of AVLT delayed recall was set at three after consulting the consortium. Even for the worst performing of the cognitively healthy participants, a score lower than three words was not expected. Such extreme scores could indicate pathology or a typing error, and therefore should be removed. A total of 217 AVLT delayed recall scores (4.5%) fell below the lower extreme border and were removed. No scores exceeded the upper extreme border.

### 2.4. Model selection

Next, we used a regression approach to remove demographically corrected outliers. Because a person's neuropsychological test scores depend to some extent on his or her demographic characteristics, not all outlying scores can be found by defining a single criterion value for all scores. For example, scores that are abnormal in young participants may not at all be abnormal in healthy elderly. To define these outliers we, therefore, first wanted to partial out the effects of age, sex, and level of education. Because the data came from multiple studies, the scores are not strictly independent. For example, some studies may give higher compensation to their participants and these may, therefore, show better scores due to higher motivation. As a second example, some studies may use more stringent exclusion criteria than other studies, and therefore may show higher scores due to the stricter selection of participants. We took variability between studies into account while estimating the effect of age, sex, and level of education using a multilevel regression approach (Curran and Hussong, 2009)^2^. The demographic variables were age in years, sex, and level of education. Level of education was coded on a seven-point scale, which is commonly used in the Netherlands (Verhage, 1964). This scale is similar to UNESCO's ISCED scale (UNESCO, 2012) on which one stands for “no education” and seven stands for “university degree.” Although this is an ordinal scale, we treated it as an interval scale and estimated the linear effect of education in order to avoid estimating separate parameters for all levels of education. To determine which effects to include, we first made a selection on the basis of how much demographic information was available, and second, a selection on the basis of which effects were statistically important enough to include in the model. These two selection steps are discussed in more detail below.

#### 2.4.1. Part 1: selection of effects based on availability of demographic data

To estimate the effects of demographic variables, a reasonable range of values on these variables is necessary. However, the range of values was narrow for some variables in the donated data. For example, for some tests only scores from higher educated people were available, which implied that the education effect for these tests could not be estimated. To find out which effects could plausibly be estimated, we tabulated age, sex, and level of education. If the median number of participants in each cell was lower than five, we considered this too sparse to estimate the corresponding effect. Because age is continuous, we temporarily created age categories, namely individuals younger than 55, aged between 55 and 75 years, and 75+.

Example: In Table 1, an example of this tabulation is given for the AVLT - delayed recall. The effect of sex is estimable, as the minimum cell count across sexes is 2249. The effect of age is considered estimable, as the median cell count across age categories is 1120. Similarly, the effect of education is considered estimable, as the median cell count across education categories is 335.

**Table 1:** **Tabulation of number of participants by sex, age categories, and level of education for the AVLT-delayed recall variable**.

**Sex N per category**	**Age N per category**	**Level of education N per category**
2249 (Men)	993 (Younger than 55)	17 (1)
2349 (Women)	2485 (55–75 year-olds)	323 (2)
Minimum: 2249	1120 (Older than 75)	119 (3)
	Median: 1120	938 (4)
		1755 (5)
		1111 (6)
		335 (7)
		Median : 335

*If the median (or minimum in the case of sex) criterion is not met for an effect, this effect cannot be included in the model*.

#### 2.4.2. Part 2: statistical selection of effects to be included in the model

Even if there are sufficient observations to estimate the effect of a demographic variable, it does not necessarily imply that the variable has an effect on the test scores. To determine which effects to include in a regression model, we used a backward selection procedure, removing effects if removal resulted in a lower Akaike Information Criterion (AIC) (Cohen et al., 2003).

**Figure 2:**
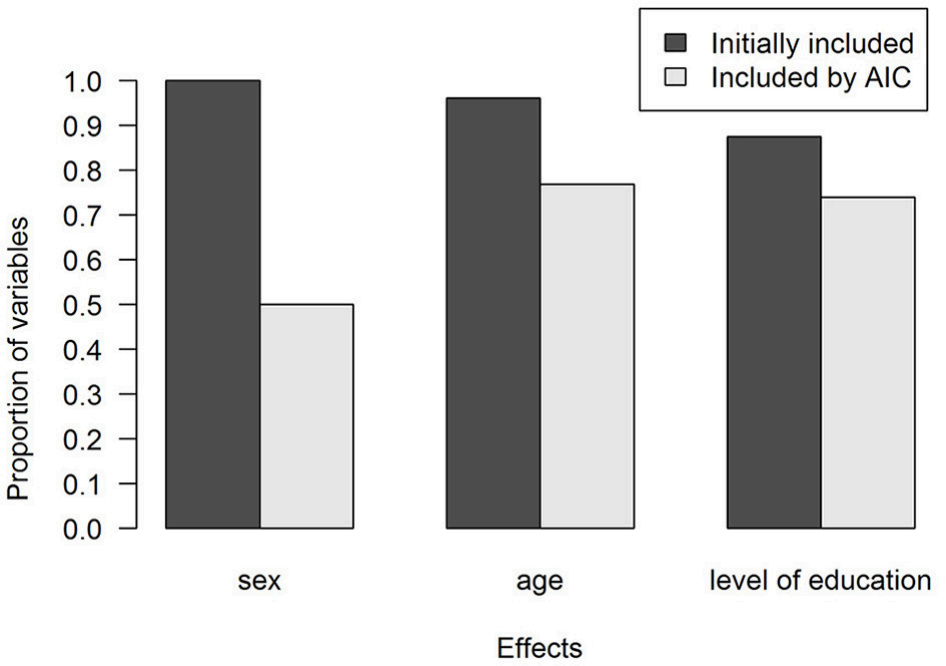
**Proportion of variables for which the demographic effects were included in the models**. In dark gray, effects that could be included after accounting for sample size constraints. In light gray, effects that were included after using the Akaike Information Criterion (AIC) to select effects.

Figure 2 shows the proportions of variables for which effects were included. As can be seen in Figure 2, there were sufficient data to estimate a sex effect for all variables, but in half of the cases, sex was found not to be predictive. Education and age effects were frequently included, if enough data were available to estimate them. The model that was selected for each variable can be found in the ANDI background documentation (www.andi.nl).

Example: For the AVLT-delayed recall the best model included all three effects.

### 2.5. Removing demographically corrected outliers

After fitting and selecting the appropriate models to correct for demographic characteristics, we used the residuals rather than the raw scores to decide whether scores were abnormal. These residuals represent the distance of the observed scores from the scores that are expected on the basis of the demographic characteristics. A common criterion for outlying values is three standard deviations from the mean. However, a few outlying scores can increase the standard deviations considerably. Therefore, we used the median absolute deviation from the median (MAD) (Leys et al., 2013), which is more robust to outliers than the standard deviation. As a cutoff criterion, we used 3.5 MAD rather than the more common three standard deviations, as we intended to include as much as possible of the distribution of normal scores. On average 0.53% of scores were removed for the 183 variables.

Example: For the AVLT-delayed recall, no scores exceeded the 3.5 MAD cut off criterion.

#### 2.5.1. Note on the removal procedure

If a participant's score on a test is outlying, one might either remove only this score, remove all of the participant's scores on this test, or remove all of the participant's scores on all tests. We opted for the first possibility, because removing scores on more variables than just the outlying one implies that we can identify the participant's cognitive functioning as the cause of the outlying value, which we cannot. The source may just as well be an administrative error.

## 3. Normality

The primary aim of the ANDI database is to facilitate normative comparisons. In both univariate and multivariate normative comparison methods, normality of the dependent variables is usually assumed (Crawford and Howell, 1998; Huizenga et al., 2007). Not all neuropsychological test scores, however, are normally distributed. This may be due to effects of demographic variables. For example, if young participants' scores are normally distributed with a low mean reaction time, and if old participants' scores are normally distributed with a high mean reaction time, then the raw scores for both groups combined may be bimodal. However, if the effect of age is partialled out in a regression analysis, and if the residual scores of this regression analysis are used instead of raw scores, such non-normality is no longer an issue. However, residual scores may still be non-normal. For example, some tests show a ceiling effect regardless of the demographic variables. In those cases, a normalizing transformation is recommended to meet the assumption of normality (Crawford et al., 2006). Scores are often transformed to normality (Jacqmin-Gadda et al., 2007) using transformations such as the square root or the reciprocal. These can both be written as power transformations, raising to the power of 0.5 and −1, respectively. Although these transformations are frequently used, they do not necessarily lead to the best approximation of normality. Therefore, we used the Box-Cox procedure (Box and Cox, 1964; Sakia, 1992) to find the best power transformation. For example, the procedure may find that the best transformation is raising to the power 0.563. The Box-Cox procedure requires a large dataset, which is not often available in neuropsychology (Crawford et al., 2006). Fortunately, the size of the ANDI database allows this Box-Cox procedure. Because in ANDI, patients will be compared to demographically corrected norms, we wanted the residuals (i.e., scores corrected for the effects of demographic variables) to be normally distributed. The algorithm therefore searches among several power transformations of the raw data (e.g., 0.506, 0.507, 0.508, etc.), and selects the power transformation resulting in the best approximation to normally distributed residuals. The power transformation that was selected for each variable can be found in the ANDI background documentation (www.andi.nl).

The Box-Cox procedure is highly flexible, but our application required a few adjustments. First, all scores have to be larger than 0. Therefore, if there were scores that were either negative or 0, a constant was added (e.g., if the greatest negative value was −5 we added the constant 5.001) to make all scores positive. Second, if the best power transformation turned out to be negative, raising the raw scores to this power flipped the order of values, i.e., the worst scores became the best and vice versa. To reverse this change of ordering, these transformed values were multiplied by −1 to restore their original order. Third, we included study as a predictor in the regression model, because we wanted the residuals to be normal within every study instead of normal over studies. Fourth, power transformations may result in tiny or huge values, which may be difficult to interpret. Therefore, we first Box-Cox transformed all scores, and then standardized all these transformed scores to the familiar *z*-scale with mean 0 and standard deviation 1. Finally, all standardized transformed *z*-scores were merged into a single dataset to create the final ANDI database.

**Figure 3:**
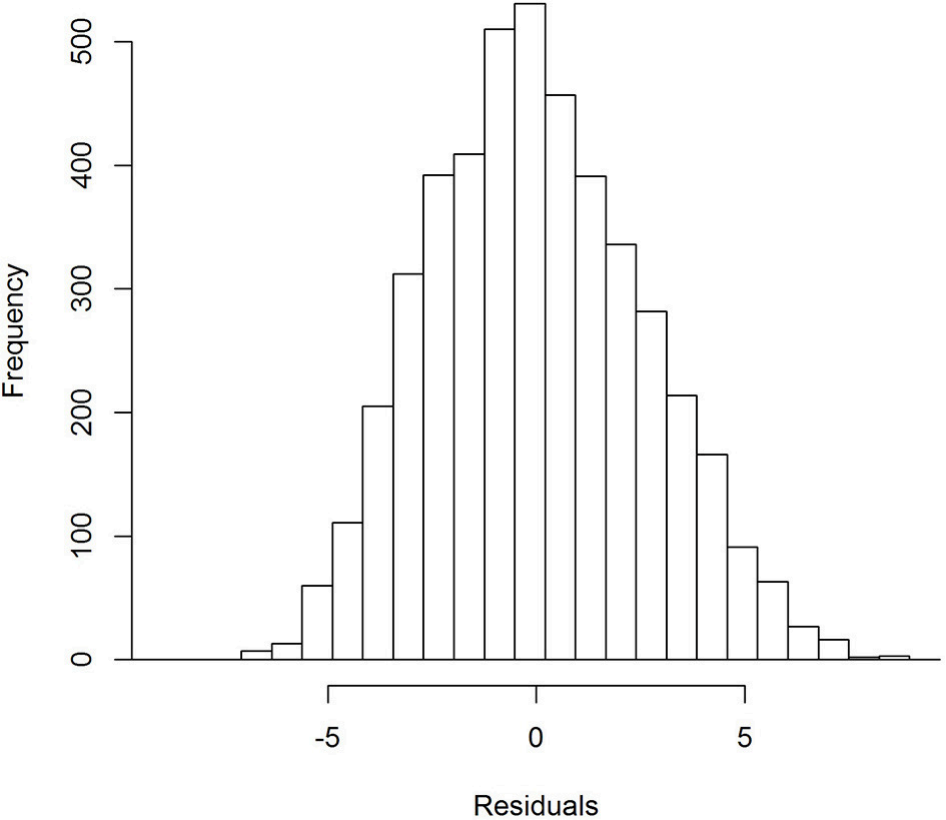
**Distribution of the residuals of the model fitted to the AVLT delayed recall variable before power transformation**.

**Figure 4:**
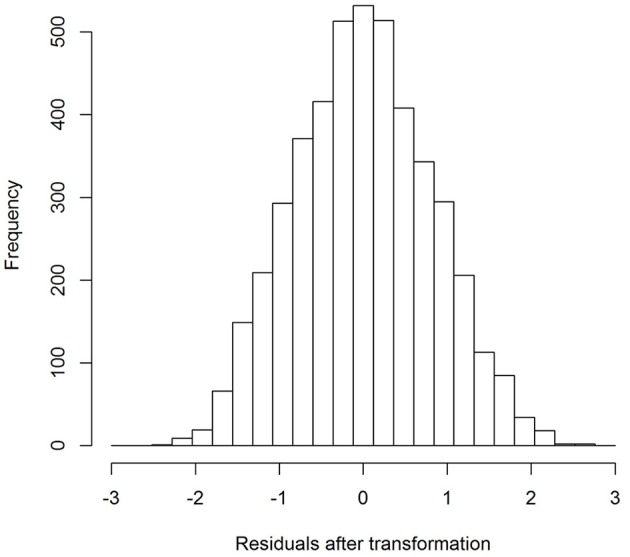
**Distribution of the residuals of the model fitted to the AVLT delayed recall variable after the power transformation of 0.75, and after standardization**.

Example: For AVLT-delayed recall, the best Box-Cox power transformation was 0.75, implying that when raw scores on AVLT-delayed recall were raised by the power 0.75, the residuals were as normally distributed as possible. In Figures 3, 4, it can be seen that the residuals were somewhat skewed before transformation and were neatly normally distributed after transformation.

When a patient's scores are compared to the scores in the database, the patient's scores are automatically transformed by the ANDI website's software using the same procedure.

### 3.1. Model evaluation

#### 3.1.1. Fit to data

After outlier removal, transformation, and standardization, the (multilevel) regression models were fitted again. This was done to get parameter estimates on the new standardized transformed scale. To evaluate whether the model fitted the raw data well, predictions from the model had to be destandardized and transformed back to the original scale. These back-transformed model predictions were plotted together with the raw data for visual inspection of model fit.

**Figure 5:**
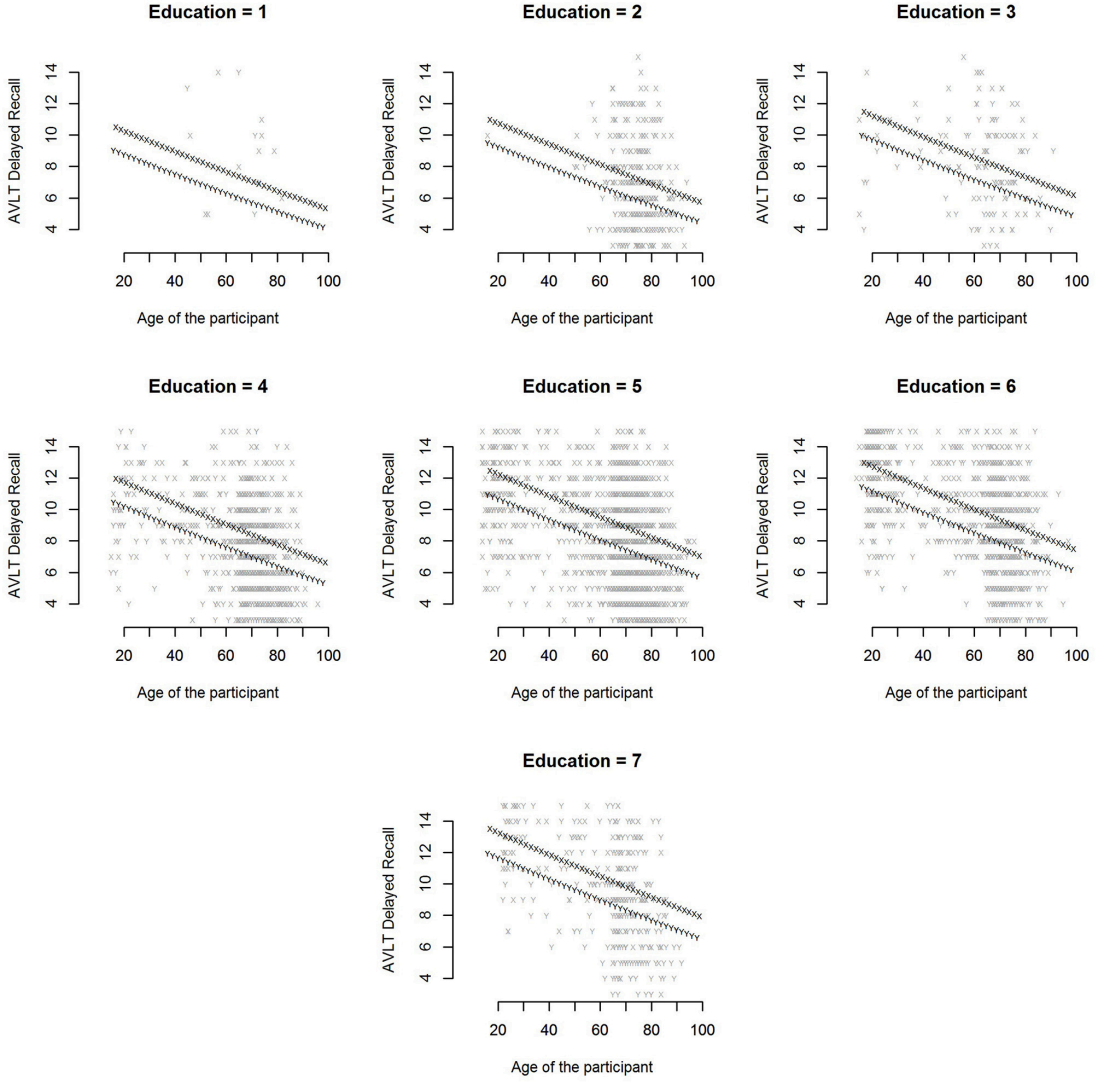
**Raw scores on the AVLT delayed recall variable are plotted against age**. Separate plots were made for the different levels of education. Men are depicted with the letter y and women with x.

Example: In Figure 5, the raw scores on the AVLT delayed recall variable are plotted as a function of age, sex, and level of education. All raw scores lie between 3 and 15, as extreme outliers have been removed. There are many data points for education levels 2 through 7, but relatively few for education level 1. All effects were included in the model. This can be observed in Figure 5. The effect of age indicates that scores decrease as participants get older. It can also be observed that men do slightly worse than women, and that scores increase with level of education.

**Figure 6:**
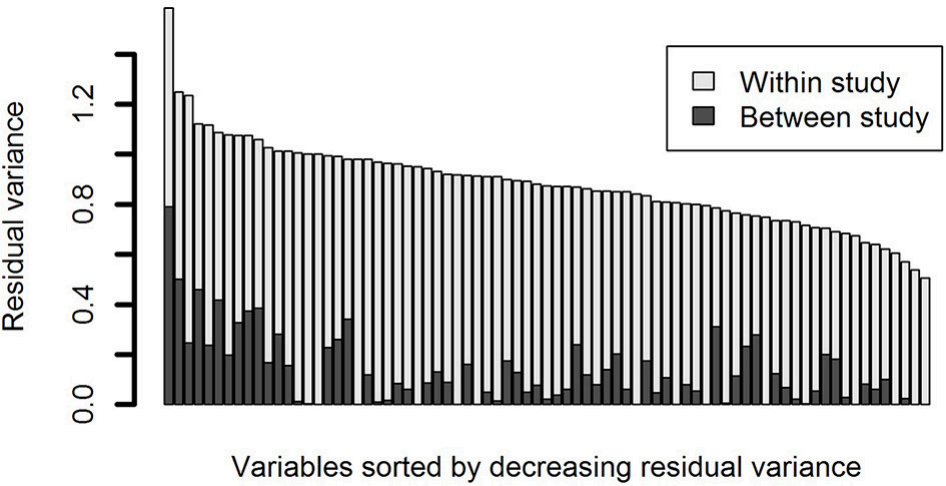
**Partitioning of total residual variance for variables that were administered in multiple studies**. Dark gray portions of the bars are the residual variance due to between study differences. Light gray portions of the bars are the residual variance due to within study/between participant differences.

In Figure 6, between and within study variance is plotted for the variables originating from multiple studies. It can be seen that between study variance exists for most of the variables, although between study variance was generally lower than within study variance.

### 3.2. Contents of ANDI

ANDI currently contains data of 26,635 healthy participants on 43 neuropsychological tests from different cognitive domains. As an example, Table 2 lists a selection of variables currently included in the database (the complete list is available on www.andi.nl).

**Table 2:** **Example variables per neuropsychological test**.

**Example variable**	**N studies**	**N in ANDI**	**Age range**	**% Men**	**Education range**
**EXECUTIVE FUNCTIONS**					
Letter Fluency (3 letters)	23	2897	17–97	48	1–7
Semantic Fluency (animals)	27	5783	17–96	40	1–7
BADS (Zoo map total)	6	398	17–86	43	1–7
**ATTENTION AND WORKING MEMORY**					
Trail Making Test (A)	37	3320	8–97	47	1–7
Trail Making Test (B)	37	3254	8–97	47	1–7
Stroop (Word card in seconds)	30	2147	16–91	43	1–7
Stroop (CW interference in seconds)	30	2132	16–91	43	1–7
**VISUOSPATIAL**					
Judgment of Line Orientation (raw score)	1	69	40–80	54	3–7
**MEMORY**					
RAVLT (delayed recall)	29	4598	14–97	49	1–7
RBMT (prose 1 delayed recall)	8	396	17–89	44	1–7
RCFT (delayed recall)	5	502	17–86	48	1–7
WAIS (Version III Coding)	9	1734	15–92	49	1–7
**LANGUAGE**					
Boston naming test (long version)	5	400	17–89	40	1–7
**INTELLIGENCE**					
Dutch adult reading test (Raw score)	26	2171	16–96	42	1–7
Raven CPM (A+B)	2	4020	55–94	48	1–7

*The number of participants, number of studies, and demographic information all refer to one example variable*.

## 4. Discussion

We described the steps to prepare the ANDI database for normative comparisons in neuropsychology. First, data were gathered from the ANDI consortium. Second, data from neuropsychological tests were merged. Third, we removed scores that could not come from cognitively healthy participants using extreme borders. Fourth, to determine for which demographic effects to correct, we selected only effects for which we had enough data and only included the effect if this was necessary according to the AIC. Fifth, after a model had been defined, we removed scores that were outlying after correction for demographic characteristics. We did this by removing scores that differed more than 3.5 MAD from the median. Sixth, because normative comparison procedures assume normality of score distributions, we used the Box-Cox procedure to search for a power transformation, that when applied to the raw data, optimally normalized the residuals after the demographic correction. These steps were applied for every variable of every neuropsychological test included in the database.

### 4.1. Benefits of the ANDI database

The ANDI database and infrastructure offer a number of advantages over existing normative data published in test manuals.

#### 4.1.1. More appropriate norms

First, the ANDI normative data have been gathered roughly in the past 20 years which make them more applicable than data that have been gathered longer ago. Because the database is internet-based, and because the ANDI construction procedure is highly automatized, it will be possible to keep the norms up-to-date by regularly adding new data and rerunning the ANDI construction procedure. Second, the ANDI database contains a considerable amount of data for old (80+) participants, making normative comparisons for this group also feasible. Third, because the data have been donated by universities and hospitals in the Netherlands and Flemish Belgium, all norms come from a population similar to patients in these countries. Fourth, scores in ANDI are corrected for the effects of age, sex, and level of education. This is an improvement over many published normative data which are typically corrected for age only. Fifth, in many traditional norms, age is not treated as a continuous variable, but is divided into arbitrary age categories. This implies that when one shifts from one age category to the next, the interpretation of the test score may change abruptly. Because in our regression approach age is treated as a continuous variable, such leaps between groups do not occur (Testa et al., 2009). Sixth, for many test variables, the ANDI norms are based on large numbers of participants (e.g., thousands) making them more precise than many existing neuropsychological norms.

#### 4.1.2. Normative comparisons with multivariate data

Another unique aspect of ANDI as a normative database is that many participants in the database have completed multiple tests. This allows multivariate normative comparisons, which have increased sensitivity to detect cognitive impairment (Huizenga et al., 2007). Multivariate norms are currently often lacking so that multivariate normative comparisons cannot be broadly applied in clinical practice. Likewise, multiple testing corrections for univariate normative comparisons which also require multivariate normative data (Huizenga et al., 2016), and normative comparisons that compare differences between test scores within one patient (Crawford and Garthwaite, 2002), become feasible. With the ANDI database and the accompanying website, such comparisons can be routinely applied.

#### 4.1.3. Exportable infrastructure

The software of the ANDI infrastructure will be freely available for researchers to be applied to other data sets. If researchers collect their own control datasets, the highly automatized procedure for merging, standardization, and correction of the scores described here could be carried out (all code is provided on https://github.com/JAvRZ/andi-dataprocessing). In this way, versions in other countries and other fields of study (such as clinical psychology or medicine) can be set up.

### 4.2. Potential limitations of the ANDI database

It is important to mention potential limitations of the ANDI database. First, ideally a normative database is based on a random sample. Although some included studies indeed sampled randomly from the population, others used convenience samples, e.g., they used family members of patients as controls. However, note that the effects of age, sex, and level of education were included in the models, thereby removing potential confounding effects of convenience sampling. Second, the sample should ideally be from a cognitively healthy population. Indeed, some donated studies assured that pathology was absent in the control sample, however others used more lenient inclusion criteria. We tried to mediate this problem by excluding impossible and outlying scores.

## 5. Concluding remark

Although our primary goal is to make a contribution to neuropsychological assessment, we also strive for broader applications. The highly automatized ANDI construction procedure software is freely available, allowing others to build their own diagnostic infrastructure. Creating such database-supported infrastructures can be an important innovation in healthcare and health research as it may provide better norms and more advanced diagnostic procedures. In research projects, it may replace collecting data from control subjects if the control data can be obtained from the database. This shows once more that data sharing has great potential. Newly created databases like ANDI provide valuable new resources while not putting any additional burden on healthy controls.

## Author contributions

NV, acquired and organized the data and wrote the manuscript. JA, developed and implemented the data analytic plan and wrote the manuscript. BS, designed the project, contacted data contributors, and revised the manuscript. JM, designed the project and revised the manuscript. HH, designed the project, developed the data analytic plan, and wrote the manuscript.

## Funding

NV and JA are supported by grant MaGW 480-12-015 awarded by the Netherlands organization for scientific research (NWO). HH is supported by grant VICI 453-12-005 awarded by NWO.

### Conflict of interest statement

The authors declare that the research was conducted in the absence of any commercial or financial relationships that could be construed as a potential conflict of interest.

## Footnotes

^1^To avoid confusion: this project is not related to ADNI, which stands for Alzheimer's Disease Neuroimaging Initiative.

^2^For variables with data from only one study, a single level regression model was fitted.
